# Predicting reattendance to the second round of the Maltese national breast screening programme: an analytical descriptive study

**DOI:** 10.1186/s12889-019-6507-9

**Published:** 2019-02-13

**Authors:** Danika Marmarà, Vincent Marmarà, Gill Hubbard

**Affiliations:** 1 Faculty of Health Sciences, University of Stirling, School of Health Sciences, Room E9, Pathfoot, Stirling, FK9 4LA Scotland; 2 Cancer Care Pathways Directorate, Sir Anthony Mamo Oncology Centre, Level -1, Dun Karm Street, Msida, MSD 2090 Malta; 3 Department of Management, University of Malta, Msida, Malta; 4 Highland Campus, Centre for Health Science, Old Perth Road, Inverness, IV2 3JH UK

**Keywords:** Breast cancer, Screening, Mammography, Attendance, Reattendance, Second invitation

## Abstract

**Background:**

A range of barriers influence women’s uptake to a first breast screening invitation. Few studies however, have examined factors associated with second screening uptake. This study follows Maltese women to explore predictors and behaviours to re-attendance, and to determine if uptake of first invitation to the Maltese Breast Screening Programme (MBSP) is a significant predictor of second screening uptake.

**Methods:**

A prospective study was conducted to determine factors associated with re-attendance for 100 women invited to the second MBSP round. Records of women’s second attendance to the MBSP were extracted in January 2016 from the MBSP database. Data were analyzed using chi-square tests, Independent Samples t-test, Mann Whitney test, Shapiro Wilk test and logistic regression.

**Results:**

There were no significant associations for sociodemographic or health status variables with second screening uptake (*p* > 0.05), except breast condition (Fisher’s exact test, *p* = 0.046). Non-attendees at second screening were most unsure of screening frequency recommendations (χ2 = 9.580, *p* = 0.048). Attendees were more likely to perceive their susceptibility to breast cancer (*p* = 0.041), believed breast cancer to be life changing (*p* = 0.011) and considered cues to action to aid attendance (*p* = 0.028). Non-attendees were in stronger agreement on mammography pain (*p* = 0.008) and were less likely to consider cues to action (15.4% non-attendees vs 1.4% attendees) (*p* = 0.017 respectively). ‘Perceived barriers’, ‘breast cancer identity’, ‘causes’ and ‘consequences’ were found to be significant predictors of second screening uptake, with ‘perceived barriers’ being the strongest. The inclusion of illness perception items improved the regression model’s accuracy in predicting non-attendance to the second screening round (84.6% vs 30.8%). First screening uptake was found to be a significant predictor of subsequent uptake (OR = 0.102; 95% CI = 0.037, 0.283; *p* = 0.000).

**Conclusions:**

Interventions to increase uptake should target first invitees since attending for the first time is a strong predictor of uptake to the second cycle. Further research is required given the small sample. Particular attention should be paid to women who did not respond to their first invite or are unsure or reluctant participants initially.

## Background

Breast cancer continues to be a major cause of female morbidity and mortality worldwide [1]. Regular breast screening (BS) for breast cancer (BC) could lead to a 25–30% reduction in mortality rates in the population of women invited for screening [2]. This evidence led to the implementation of screening programmes across Europe based on the EU Council recommendations, which recommend biennial screening mammography in average-risk women aged 50–69 years [3]. A Maltese breast screening programme (MBSP) was set up in Malta in 2009 to screen women (aged 50 – 60 years) through mammography every 3 years [4], aiming gradual expansion to reach women until the age of 69 years while reducing the screening time interval as in other countries [5]. In 2015, this age cohort (50–60 years) was in its second BS round.

Breast screening uptake is defined as “the proportion of women invited who attend for screening within 6 months of their invitation” ([6], p.1). In Malta, the overall attendance rate was below the acceptable target of 70% in the first BS round [7]. Similarly, substantial variation in BC uptake exists across other countries [1]. An important factor that merits exploration includes regular screening adherence, because of its significant impact on morbidity and mortality reductions [8, 9]. Detecting BC early is not ensured by a one-off BS attendance [10], but on the consistency of attendance in line with recommended time intervals [11]. The literature suggests that previous mammography use is highly associated with future use [12] because women believe in the effectiveness of screening which in turn increases their intentions to go for screening, resulting in their adherence to subsequent screens [13]. An earlier study by Cockburn and colleagues [14] found that those having weakest intentions to attend for their first screening are less likely to attend for their second screening (OR = 0.44, CI 0.23, 0.85). Therefore, it has been suggested that programs should focus on reaching those who have underutilized mammography in their past [12] as this would feed into attendance in subsequent BS invitation rounds. Nonetheless, many studies have focused on the reasons for one-time screening rather than subsequent use [9, 15] and limited studies have sought to specifically explore predictors of uptake to *second* round BS invitations [14, 16, 17]. In a comparative study of 200 re-attenders and 200 non-re-attenders for second triennial National Breast Screening Programme appointments in Nottingham [10], the 200 women who failed to accept their invitation implicated their negative initial screening experience in their decision, with 41% implicating pain, 6% stress and 3% embarrassment.

To our knowledge, no research yet exists on women’s reattendance at the MBSP or on screening predictors to the second BS round in Malta. In 2017, we reported on first screening uptake of the MBSP [4]. We found that Health Belief Model (HBM) constructs were the strongest predictors of uptake of first invitation to the MBSP, though the inclusion of illness representation dimensions improved the predictive accuracy for non-attendance. In the present study, we follow a sub-sample of Maltese women with three main objectives: (1) to determine their re-attendance at the second BS invitation round; (2) to explore whether sociodemographic factors, health status, knowledge, health beliefs and illness perceptions are significant predictors of second BS uptake; and (3) to determine if uptake of first invitation to attend the MBSP is a significant predictor of uptake to the second invitation in Malta.

## Theoretical framework

BS behaviour is influenced by a number of factors, including health beliefs [8], illness representations [8], knowledge of BC and BS [4], socio-demographic factors [4], and health status (medical factors) [8]. These factors were found to influence BS attendance in the first invitation round in Malta [4]. The Health Belief Model (HBM) was used as a theoretical framework for this study, due to its application in myriad preventive health behaviours, including BS [8, 18–20]. The HBM consists of six main variables (constructs), namely perceived susceptibility, perceived severity, perceived benefits, perceived barriers, cue to action, and self-efficacy [19]. These variables play an important role in an individual’s perception about BS, such that women are more likely to perform BS if they feel susceptible to BC or the risks of contracting BC (perceived susceptibility), believe in the seriousness and consequences of the disease (perceived severity), perceive more benefits than barriers from undergoing mammography, have higher confidence for obtaining mammography, and take heed in cues to action [19]. Based on the HBM, women’s perceptions about BC are derived from their knowledge and perceptions about the disease, which predicts whether women will attend for mammography [18]. However, the HBM does not include the impact of emotions to predict BS behaviours, which is why the Common-Sense Model (CSM) [20], also used to explain BS behaviour in a small number of studies [8, 21–25], was utilised in this study. The CSM considers the cognitive and emotional representations of a health threat [8, 26, 27] and guides subsequent coping behaviour [28]. Illness perceptions comprise the following constructs: *BC identity, cause, cancer timeline (acute/chronic, cyclical), consequences, personal and treatment control, illness coherence and emotional representations.* [8, 21].

## Methods

### Study design and setting

The full details of the methods are described in detail in our prior paper [4]. This prospective study was conducted in Malta in January 2016 to determine factors associated with the second BS cycle.

### Participants

Of the 404 women surveyed between June and September 2015 about their first BS attendance [4], 100 women were identified in January 2016 to have subsequently been invited to the second round. These 100 women were a sub-sample of participants of our larger study [4] who had received an invitation to attend both the first and second MBSP round. Description and characteristics of the larger sample (*n* = 404) are found in the previous study [4].

### Measures

To predict reattendance, we used our previously constructed 121-item questionnaire based on Champion’s Health Belief Model Scale (CHBMS-MS) and the Revised Illness Perception Questionnaire (IPQ-R). The full details of its translation, adaptation and pilot-testing among Maltese women have been described in detail in our prior published paper [29].

### Variable definitions

A second invitation was defined as the second (subsequent) time a woman is invited to the MBSP and either attends or does not attend for the mammogram. Women were considered eligible in this study if their scheduled appointment date had elapsed for their second BS invitation and they had not informed the unit to reschedule their mammography invitation. A screening invitation is posted to the client approximately 1 month before the scheduled mammography date. Hence, those women invited to the second BS round and awaiting their scheduled day for mammography screening were not considered in this study.

### Data analysis

Several statistical tests were used to analyse the differences between attendance or non-attendance to the second MBSP invitation. Chi-square test was used to test for association between two categorical variables (e.g. attendance against women’s knowledge of the breast screening frequency); however, Fisher’s exact test was used when the assumptions of the Chi-Square test were violated. The Shapiro-Wilk test was used to test for normality of all construct variables. Furthermore, the following tests were used throughout the analysis: the independent samples t-test and the non-parametric Mann-Whitney test. Significant predictors for re-attendance were determined through the binary logistic regression. Hence, several models were developed (using different constructs and variables) to predict attendance and non-attendance to the second MBSP invitation. The ‘backward-elimination’ was applied to exclude any non-significant variables from the latter models and the final output included the unstandardized coefficients, standard error, the Wald value, *p*-values, Odds Ratios (ORs) and 95% confidence intervals (95% CIs). The data was analyzed using SPSS version 21 under the guidance of an expert statistician. A *P*-value of < 0.05 was considered statistically significant (Table 1).

**Table 1:** Sample Characteristics (*n* = 100)

Characteristics	Mean	SD	N	%
Age (year)	58.63	2.63		
Education level				
Primary level			26	26.0
Secondary level			68	68.0
Tertiary level			6	6.0
Occupation				
Pensioner			4	4.0
Housewife			78	78.0
Employee			18	18.0
Status				
Single			4	4.0
Married			85	85.0
Separated/Divorced			5	5.0
Widowed			6	6.0
Family income				
Less than €10,737			28	28.0
€10,737 – €16,113			32	32.0
€16,114 – €23,563			8	8.0
€23,564 – €33,966			2	2.0
Greater than €33,966			2	2.0
Prefer not to say			30	30.0
Own a car				
Yes			78	78.0
No			22	22.0
Drive				
Yes			46	46.0
No			54	54.0
Any illness, disability or condition				
Yes			58	58.0
No			42	42.0
Family physician (GP)				
Yes			92	92.0
No			8	8.0
Frequency of GP visit				
Only when I have a problem			80	80.0
Once a month			5	5.0
More than once a year			7	7.0
Missing			8	8.0
Lumpy breasts				
Yes			9	9.0
No			91	91.0
Relatives or close friends had cancer				
Yes			89	89.0
No			9	9.0
Prefer not to say			2	2.0

## Results

### Sample characteristics

Table 1 presents the sample characteristics (*n* = 100). At the time of the second screening cycle, women were aged between 53 and 63 years with a mean of 58.63 years (±2.63 SD (standard deviation)).

**Fig. 1:**
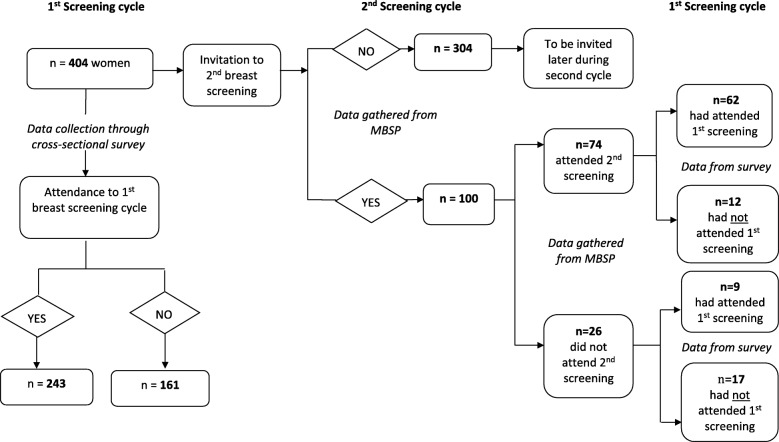
First and second breast screening invitation pathway

From the 100 women invited to the second screening round (Fig. 1), nearly three-quarters of this sample (74%; *n* = 74) attended the second screening round; of these, 83.8% (*n* = 62) had responded to their first invitation while 16.2% (*n* = 12) had not attended the first screening round. Over a quarter of our sample (26%; *n* = 26) did not attend the second screening round; of these, 34.6% (*n* = 9) had attended the first screening cycle while 65.4% (*n* = 17) had not responded to their first invitation. Women who attended their first invitation were more likely to sustain screening mammography adherence than non-attendees (χ2 = 22.6, *p* < 0.001).

## Associations between psychosocial factors, and attendance and non-attendance to a second BS invitation

Similar to the analysis carried out in our previous papers to analyse the associations between attendees and non-attendees in relation to lifetime mammography practices [30] and timely adherence [31], the following analysis focused on the associations between attendees and non-attendees to a second BS invitation.

### Sociodemographic factors and health status

There was a significant association between breast condition (lumps or cysts but not BC) and second screening uptake (*Fisher’s exact test* applied: *p* = 0.046), whereby from those who attended their second invitation, 15.1% had a breast condition, whereas from among non-attendees, 34.6% had a breast condition. There were no significant associations for other sociodemographic or health status variables (*p* > 0.05).

### Knowledge

There was a significant association between knowledge about recommended frequency for attending BS; women who did not attend the second screening invitation were most unsure of the recommended frequency (χ2 = 9.580, *p* = 0.048).

### Health beliefs

No statistical significance was found for the majority of the HBM constructs, except for 5 items (Table 2) as follows: 25.7% of women who attended the second round strongly disagreed with the statement: ‘there is no is possibility of getting breast cancer’ as opposed to 11.5% of non-attendees (*p* = 0.041), while 23.1% of non-attendees were undecided. Attendees considered more strongly than non-attendees (41.9% vs 19.2%) that they would sustain mammography adherence if their GP advised them to attend (*p* = 0.028). Non-attendees were in stronger agreement than attendees (11.5% vs 0.0%) that mammography is painful or uncomfortable (*p* = 0.008). Non-attendance at the second invitation round was more likely for women who considered reminder letters, reminder phone calls and text messages not to be of help (15.4% non-attendees vs 1.4% attendees) (*p* = 0.017 respectively).

**Table 2:** Comparison of Health Beliefs between attendees and non-attendees

Attendance to the second breast screening invitation	Yes		No		Total		Chi-Square test^a^	
Health Beliefs	Mean	SD	Mean	SD	Mean	SD	χ2	*p*-value
There is no possibility of getting breast cancer *(r)*	1.8	0.6	2.2	0.7	1.9	0.6	0.3	0.041*
Your chances of getting breast cancer are high	3.6	0.7	3.5	0.7	3.6	0.7	1.1	0.789
There may be the possibility of developing breast cancer in your lifetime	4.0	0.3	3.9	0.4	4.0	0.3	3.8	0.147
When you get a mammogram, you feel good about yourself	4.0	0.3	3.9	0.5	4.0	0.4	6.5	0.088
When you get a mammogram, you do not worry as much about breast cancer	3.8	0.7	3.7	0.7	3.8	0.7	1.2	0.759
Having a mammogram will help you find lumps early in your breasts	4.2	0.5	4.1	0.5	4.2	0.5	3.3	0.355
If you find a lump through a mammogram, the treatment for breast cancer may not be as bad	4.0	0.4	4.0	0.2	4.0	0.3	1.2	0.750
Having a mammogram will decrease your chances of dying from breast cancer	4.0	0.4	3.9	0.4	4.0	0.4	4.3	0.227
Having a mammogram will help you find a lump before it can be felt by yourself or a health professional	4.0	0.4	3.9	0.4	4.0	0.4	3.3	0.344
Having a routine mammogram would make you anxious about breast cancer	2.7	1.0	3.0	1.1	2.8	1.0	1.6	0.449
Having a routine mammogram would make you worry	2.7	1.0	2.9	1.1	2.7	1.0	1.5	0.685
You fear having a mammogram because you might find out that something is wrong	2.8	1.1	3.2	1.2	2.9	1.1	6.2	0.103
You fear having a mammogram because you do not know the procedure or what to expect	2.1	0.5	2.4	0.8	2.2	0.6	4.8	0.092
You fear having a mammogram because you know someone (family or friend) with breast cancer	2.6	1.1	2.7	1.2	2.6	1.1	0.6	0.907
It is embarrassing for you to have a mammogram	2.3	0.8	2.4	0.9	2.4	0.8	0.5	0.779
Undergoing mammography will be painful or uncomfortable	3.2	1.0	3.3	1.1	3.2	1.0	11.9	0.008*
Having a mammogram is time consuming	1.3	0.5	1.3	0.7	1.3	0.5	3.8	0.153
You are discontent with Breast Screening personnel as they have been rude to you	1.1	0.4	n/a	n/a	1.1	0.4	n/a	n/a
You have fear or distrust in the medical team	1.7	0.8	2.0	0.8	1.8	0.8	4.7	0.094
Having a mammogram would expose you to unnecessary radiation	2.2	0.6	2.4	0.8	2.3	0.7	1.6	0.652
You have too many other problems in your life than to get a mammogram done	1.7	0.6	1.8	0.7	1.7	0.6	0.7	0.699
You are not old enough to have a mammogram periodically	1.8	0.4	1.8	0.4	1.8	0.4	0.9	0.344
If your GP advises you to attend for a mammogram, you will attend	4.4	0.5	4.1	0.6	4.3	0.6	9.1	0.028*
If your relatives or friends advise you to attend for a mammogram, you will attend	3.6	0.9	3.3	1.0	3.5	0.9	1.9	0.586
If someone close to you has been diagnosed with breast cancer, you will attend for a mammogram	4.3	0.9	4.2	0.9	4.3	0.9	0.9	0.826
Hearing about breast cancer and breast screening in the media or news makes you think about getting a mammogram	3.6	0.8	3.4	1.0	3.6	0.9	1.7	0.418
Reminder letters would help you to get a mammogram	4.0	0.3	3.7	0.8	3.9	0.5	8.1	0.017*
Reminder phone calls or text messages would help you to get a mammogram	4.0	0.3	3.7	0.8	3.9	0.5	8.1	0.017*
Routine educational talks regarding breast cancer awareness would help you to get a mammogram	3.6	0.8	3.4	1.0	3.6	0.9	1.7	0.418
You feel confident that if you had a mammogram done, any abnormalities in your breasts will be detected	3.7	0.6	3.5	0.7	3.7	0.6	3.1	0.378
You can arrange other things in your life to get a mammogram	4.2	0.6	4.0	0.9	4.1	0.7	6.2	0.103
In case you need a mammogram, you will find a place to get it done	4.2	0.5	4.1	0.5	4.2	0.5	3.3	0.355
You can make an appointment for a mammogram	4.2	0.5	4.2	0.5	4.2	0.5	3.3	0.355
You can arrange transportation to get a mammogram	4.2	0.5	4.2	0.5	4.2	0.5	3.3	0.355
You can talk to people at the breast screening centre about your concerns	4.1	1.0	n/a	n/a	4.1	1.0	n/a	n/a
You can find a way to pay for a mammogram if you need to	4.3	0.4	4.2	0.5	4.2	0.5	2.9	0.234

*R* Reverse scored

*Significant at α = 0.05

^a^Chi-square test was applied for all health beliefs; hence the categorical answers were used to apply this test for association. For each question, respondents were asked to select a number between 1 and 5, where 1 = strongly disagree and 5 = strongly agree

### Illness perceptions

No statistical significance was found for all illness perception items except for one variable (Table 3): those who attended the second screening round agreed more strongly than non-attendees (39.2% vs 7.7%) that their whole life would change if breast cancer occurred (*p* = 0.011).

**Table 3:** Comparison of Illness Perceptions between attendees and non-attendees

Attendance to the second breast screening invitation	Yes		No	Total			Chi-Square test^a^	
Illness Perception	Mean	SD	Mean	SD	Mean	SD	χ2	*p*-value
The presence of a lump or thickening in the breast	3.9	0.3	3.9	0.4	3.9	0.4	4.8	0.186
Nipple discharge	3.9	0.4	3.8	0.4	3.9	0.4	0.8	0.666
Sudden nipple retraction	3.8	0.4	3.7	0.5	3.8	0.4	1.3	0.509
Change in shape or appearance of the nipple	3.9	0.3	3.8	0.4	3.9	0.3	1.0	0.603
Breast swelling, dimpling, redness or soreness of the skin	3.9	0.3	3.7	0.5	3.9	0.4	4.2	0.124
Skin changes of the breast	3.8	0.4	3.7	0.5	3.8	0.4	1.3	0.509
A sudden change in breast size	3.9	0.4	3.8	0.4	3.9	0.4	3.4	0.180
Aching breasts	3.6	0.6	3.4	0.6	3.6	0.6	4.1	0.132
Stress or worry	2.9	0.9	2.8	0.8	2.9	0.9	0.8	0.673
Your mental attitude (e.g. thinking about life negatively)	2.4	0.6	2.2	0.5	2.4	0.6	1.9	0.379
Family problems or worries	2.9	0.9	2.7	0.8	2.8	0.9	1.5	0.477
Overwork	2.4	0.7	2.2	0.6	2.4	0.7	1.5	0.483
Your emotional state (e.g. feeling down, lonely, anxious, empty)	2.5	0.8	2.3	0.5	2.5	0.7	3.6	0.167
Your personality	2.5	0.8	2.3	0.5	2.4	0.7	1.9	0.597
Hereditary - it runs in the family	4.6	0.5	4.4	0.7	4.6	0.6	5.1	0.080
Diet or eating habits	3.3	0.9	2.8	0.9	3.2	0.9	7.1	0.069
Poor medical care in the past	3.3	0.9	3.3	0.8	3.3	0.9	0.9	0.818
Your own behaviour	2.6	0.7	2.7	0.6	2.6	0.7	3.6	0.164
Ageing	3.0	0.9	3.2	0.9	3.1	0.9	1.2	0.562
Smoking	3.7	0.6	3.5	0.8	3.7	0.7	3.5	0.174
Alcohol	3.5	0.8	3.2	0.9	3.4	0.8	2.6	0.272
A germ or virus	3.1	0.8	3.0	0.8	3.1	0.8	0.8	0.686
Pollution in the environment	3.7	0.7	3.3	1.0	3.6	0.8	6.2	0.104
Altered immunity	3.6	0.7	3.8	0.5	3.7	0.6	5.7	0.129
Chance or bad luck	2.9	1.0	3.1	1.0	2.9	1.0	4.1	0.386
Accident or injury	3.0	1.0	3.0	0.9	3.0	0.9	0.9	0.831
Breast cancer will last a short time	2.7	0.7	2.7	0.5	2.7	0.7	3.8	0.286
Breast cancer is likely to be permanent rather than temporary	3.4	0.7	3.2	0.8	3.3	0.7	3.4	0.178
A patient with breast cancer goes through cycles in which her illness gets better and worse	3.6	0.7	3.4	0.7	3.5	0.7	3.0	0.394
Breast cancer has major consequences on a patient’s life	4.4	0.6	4.1	0.6	4.3	0.6	6.3	0.096
Breast cancer will not have much effect on your life	1.5	0.7	1.5	0.8	1.5	0.7	3.7	0.297
Breast cancer would strongly affect the way others see you	3.4	0.9	3.6	0.8	3.5	0.8	1.4	0.506
Breast cancer has serious economic and financial consequences	3.9	0.5	3.8	0.5	3.9	0.5	1.9	0.587
Breast cancer would strongly affect the way you see yourself as a person	4.1	0.5	4.1	0.6	4.1	0.5	0.6	0.736
Breast cancer would threaten a relationship with your husband or partner	3.1	1.0	2.9	0.9	3.1	0.9	1.4	0.714
If you had breast cancer, your whole life would change	4.4	0.6	4.0	0.4	4.3	0.6	11.2	0.011*
If you developed breast cancer, the chances of living a long life would decrease	4.1	0.4	4.0	0.0	4.0	0.3	3.9	0.142
There is a lot which you can do to control the symptoms if Breast Cancer occurs	3.9	0.5	4.0	0.0	3.9	0.4	1.8	0.604
The course of Breast Cancer will depend on your actions	4.0	0.4	4.0	0.0	4.0	0.3	1.5	0.691
Your actions will have an effect on the outcome of Breast Cancer	4.0	0.4	4.0	0.0	4.0	0.3	2.2	0.524
There is no treatment that will help to improve Breast Cancer	2.1	0.6	2.0	0.6	2.0	0.6	0.5	0.930
The treatment provided will be effective in controlling or curing Breast Cancer	4.0	0.2	4.0	0.2	4.0	0.2	0.4	0.804
The negative effects of Breast Cancer can be prevented or avoided by the treatment given	4.0	0.2	4.0	0.2	4.0	0.2	1.0	0.621
You have a clear picture and understanding of Breast Cancer	3.6	0.7	3.7	0.7	3.7	0.7	0.7	0.722
Breast Cancer is a mystery to you	3.2	1.0	3.2	1.0	3.2	1.0	1.3	0.860
You get anxious when you think about Breast Cancer	3.6	1.1	3.7	1.0	3.6	1.1	3.2	0.359
Breast Cancer makes you feel afraid	4.2	0.7	4.3	0.5	4.2	0.7	2.2	0.523
You get worried when you think about Breast Cancer	4.3	0.8	4.4	0.5	4.3	0.7	2.3	0.504

*Significant at α = 0.05

^a^Chi-square test was applied for all illness perceptions; hence the categorical answers were used to apply this test for association. For each question, respondents were asked to select a number between 1 and 5, where 1 = strongly disagree and 5 = strongly agree

## Differences between attendees and non-attendees

We further explored health beliefs and illness perceptions as ‘constructs’ and which of these constructs were statistically significant with second BS uptake. Table 4 shows the relationship between all 14 constructs respectively (*perceived susceptibility, perceived benefits, perceived barriers, cues to action, self-efficacy, breast cancer identity, causes of breast cancer, cancer timeline: acute/chronic, cancer timeline: cyclical, consequences, personal control, treatment control, illness coherence, emotional representations),* and attendance and non-attendance to second BS round.

**Table 4:** Comparisons between health beliefs/illness perception constructs and second breast screening uptake

	Range	Attendees (*n* = 74)	Non-attendees (*n* = 26)	Test Statistic	*p*-value
Perceived Susceptibility	3 – 15	M = 9.5, SD = 0.8	M = 9.6, SD = 0.9	1019.5^b^	0.626
Perceived Benefits	6 - 30	M = 24.0, SD = 1.7	M = 23.6, SD = 1.6	864.0^b^	0.384
Perceived Barriers	13 – 65	M = 27.0, SD = 4.5	M = 29.2, SD = 6.1	-2.0^a^	0.049*
Cues to action	7 – 35	M = 27.6, SD = 2.8	M = 25.9, SD = 4.4	726.5^b^	0.061
Self-Efficacy	7 – 35	M = 24.9, SD = 2.7	M = 24.2, SD = 2.7	783.0^b^	0.129
Breast Cancer Identity	8 – 40	M = 30.8, SD = 1.9	M = 29.9, SD = 2.5	776.0^b^	0.124
Causes of Breast Cancer	18 – 90	M = 55.8, SD = 7.2	M = 53.9, SD = 5.9	1.2^a^	0.238
Cancer Timeline: Acute/Chronic	2 – 10	M = 6.1, SD = 0.8	M = 5.9, SD = 0.8	839.0^b^	0.295
Cancer Timeline: Cyclical	1 – 5	M = 3.6, SD = 0.7	M = 3.4, SD = 0.7	829.5^b^	0.221
Consequences	8 – 40	M = 28.8, SD = 2.3	M = 28.1, SD = 1.9	744.0^b^	0.083
Personal Control	3 – 15	M = 11.9, SD = 0.7	M = 12.0, SD = 0.0	1014.0^b^	0.432
Treatment Control	3 – 15	M = 10.0, SD = 0.6	M = 9.9, SD = 0.6	865.0^b^	0.265
Illness Coherence	2 – 10	M = 6.8, SD = 1.2	M = 6.9, SD = 1.1	991.5^b^	0.802
Emotional Representations	3 – 15	M = 12.1, SD = 2.3	M = 12.4, SD = 1.8	991.5^b^	0.811

*Significant at α = 0.05

^a^ Independent Samples t-test

^b^ Mann Whitney

The ‘perceived barrier’ construct was the only statistically significant variable that described the variance between attendees and non-attendees.

## Predictors of attendance and non-attendance to the second screening round

Similar to the analyses carried out in our previous paper to predict lifetime mammography attendance in Malta [30], the following analyses focused on predicting attendance and non-attendance to the second BS invitation. The most significant variables for all logistic regression models are presented in Table 5. Logistic regression models 1 and 2 incorporated all items related to demographic and health status variables respectively, though none were found to be significant predictors of second BS uptake.

**Table 5:** Logistic Regression Models on second breast screening uptake against different variables and different constructs

	B	SE	Wald	*P*-value	OR	95% CI	Model Accuracy YES	Model Accuracy NO
Model 1: Demographics							100%	0%
Age	-0.154	0.084	3.329	0.068	0.858	0.727, 1.011		
Constant	7.926	4.905	2.611	0.106	2769.527			
Model 2: Health Status							100%	0%
Breast condition	-1.093	0.526	4.315	0.038	0.335	0.119, 0.940		
Constant	0.893	0.940	0.902	0.342	2.441			
Model 3: Health Beliefs							93.2%	30.8%
No possibility of getting breast cancer	1.064	0.474	5.030	0.025	2.897	1.144, 7.338		
Fear of unknown procedure^a^	0.563	0.388	2.102	0.147	1.755	0.820, 3.756		
GP advice	-1.145	0.562	4.158	0.041	0.318	0.106, 0.956		
Constant	0.480	2.717	0.031	0.860	1.617			
Model 4: Illness Perceptions							89.2%	69.2%
Breast swelling, dimpling, redness or soreness of the skin	-1.796	0.720	6.215	0.013	0.166	0.040, 0.681		
Diet	-1.029	0.312	10.873	0.001	0.357	0.194, 0.659		
Altered immunity	1.462	0.568	6.610	0.010	4.313	1.415, 3.141		
Whole life would change	-1.334	0.533	6.257	0.012	0.263	0.093, 0.749		
Constant	9.082	3.931	5.337	0.021	8796.855			
Model 5: Health Beliefs and Illness Perceptions							89.2%	69.2%
Breast swelling, dimpling, redness or soreness of the skin	-1.796	0.720	6.215	0.013	0.166	0.040, 0.681		
Diet	-1.029	0.312	10.873	0.001	0.357	0.194, 0.659		
Altered immunity	1.462	0.568	6.610	0.010	4.313	1.415, 3.141		
Whole life would change	-1.334	0.533	6.257	0.012	0.263	0.093, 0.749		
Constant	9.082	3.931	5.337	0.021	8796.855			
Model 6: Health Beliefs and Illness Perceptions							95.9%	84.6%
Early detection	5.699	2.097	7.390	0.007	298.646	4.904, 18,187.040		
If early detection, treatment not as bad^b^	12.267	7.293	2.830	0.093	2.126 × 10^5^	0.132, 3.427 × 10^11^		
Having mammography decreases chances of dying^b^	-8.890	6.724	1.748	0.186	0.000	0.000, 72.821		
Fear of unknown procedure	5.210	1.842	8.003	0.005	183.103	4.955, 6765.914		
Unnecessary radiation	4.471	1.655	7.301	0.007	87.419	3.414, 2238.732		
Breast swelling, dimpling, redness or soreness of the skin	-8.961	3.119	8.252	0.004	0.000	0.000, 0.058		
Personality	-5.566	2.295	5.885	0.015	0.004	0.000, 0.343		
Diet	-5.558	1.946	8.160	0.004	0.004	0.000, 0.175		
Germ or virus	-5.721	2.168	6.967	0.008	0.003	0.000, 0.229		
Altered immunity	8.217	2.860	8.254	0.004	3705.048	13.620, 1.008 × 10^6^		
Breast cancer last short time	-2.623	1.042	6.340	0.012	0.073	0.009, 0.559		
Affects the way others see you	3.105	1.286	5.831	0.016	22.305	1.795, 277.210		
Whole life would change	-9.738	3.266	8.888	0.003	0.000	0.000, 0.036		
You get worried if breast cancer occurs	2.444	1.199	4.159	0.041	11.521	1.100, 120.694		
Constant	14.947	26.502	0.318	0.573	3.102 × 10^6^			
Model 7: The 14 constructs							94.6%	30.0%
Perceived barriers	0.155	0.054	8.305	0.004	0.167	1.051, 1.296		
Breast cancer identity	-0.231	0.120	3.691	0.055	0.794	0.627, 1.005		
Causes of breast cancer^c^	-0.070	0.040	3.022	0.082	0.932	0.861, 1.009		
Consequences^c^	-0.204	0.116	3.082	0.079	0.815	0.649, 1.024		
Constant	11.286	4.983	5.129	0.024	79,721.454			

*B* Unstandardized coefficients, *SE* Standard error, *OR* Odds ratio, *CI* Confidence interval

^a^‘Fear of unknown procedure’ was retained due to better accuracy in the logistic regression model. Without this variable, the accuracy would decrease from 30.8 to 19.2%

^b^‘If early detection, treatment not bad’ and ‘Having mammography decreases death’ were retained due to better accuracy in the logistic regression model. Without these variables, the accuracy would decrease from 84.6 to 76.9%

^c^‘Causes of breast cancer’ and ‘Consequences’ were retained due to better accuracy in the logistic regression model. Without these variables, the accuracy would decrease from 30.0 to 15.4%

Models 3 to 7 include the psychosocial variables. Model 3 incorporated all HBM variables, of which three variables were found to be good predictors of second BS uptake: ‘there is no possibility of getting breast cancer’, ‘fear of the unknown procedure’, and ‘GP advice to attend’ (Table 5). For this model, attendance was predicted with an accuracy of 93.2% and non-attendance with 30.8% accuracy. When removing ‘fear of the unknown procedure’ from the model, the accuracy decreased from 30.8 to 19.2% and hence was retained even though *p* > 0.05.

Model 4 included all IPQ-R variables, of which four IPQ-R variables were found to be good predictors: ‘breast swelling, dimpling, redness or soreness of the skin’, ‘diet’, ‘altered immunity’, and ‘if you had BC, your whole life would change’ (Table 5). The accuracy for this model was 89.2% for attendance and 69.2% for non-attendance.

Model 5 focused on the significant variables found in model 3 and 4 together; i.e. on 12 variables (five Health Beliefs and seven Illness Perception variables). The final model (Model 5) retained the same significant predictors as in Model 4, excluding the HBM variables, hence showing that illness perceptions are important predictors for second BS uptake. The model accuracy, when combining both scores, was identical to that of Model 4, predicting attendance by 89.2% and non-attendance by 69.2%.

Model 6 incorporated all individual Health Belief and Illness Perception items, of which 14 variables were significantly different. However, the latter variables made this model more complex due to the large number of predictors. The model accuracy improved substantially to 95.9% for attendees and 84.6% accuracy for non-attendees. The model accuracy decreased from 84.6 to 76.9% when the following variables were removed from the model: ‘if you find a lump through a mammogram, the treatment for breast cancer may not be as bad’ and ‘having a mammogram will decrease your chances of dying from breast cancer’. Hence, the latter variables were retained even though *p* > 0.05.

A logistic regression model (Model 7) was constructed with all 14 constructs (i.e. HBM and IPQ-R as constructs not individual items). The following constructs were the most significant predictors of second screening uptake: ‘perceived barriers’, ‘breast cancer identity’, ‘causes of breast cancer’ and ‘consequences’. The ‘perceived barriers’ construct was found to be the strongest predictor. However, non-attendance was predicted with an accuracy of 30.0%, which is inferior when compared to Models 5 and 6 (69.2%). Moreover, when removing the constructs ‘causes of breast cancer’ and ‘consequences’ from the model (*p*-value slightly greater than 0.05), the accuracy to predict non-attendance would decrease from 30.0 to 15.4% even though *p* > 0.05.

Although ‘perceived barriers’ remains the most important construct to describe the variance between attendees and non-attendees, illness perception constructs (breast cancer identity, its causes and consequences) can also be considered as strong predictors of second BS uptake; a result further echoed in Model 5, where the predictors are all related to illness perceptions (breast cancer identity and consequences). Hence, although Health Beliefs are important predictors of BS uptake, the model accuracy improved with the inclusion of illness perception items into one logistic regression model (Model 6 vs Model 3).

## Predicting attendance to second screening using first screening uptake

When a logistic regression model was applied to predict second (subsequent) BS uptake using the first BS uptake as the predictor (Table 6, Model 8), non-attendance was predicted with an accuracy of 65.4% and attendance was predicted with an accuracy of 83.8%.

**Table 6:** Logistic regression analysis on the prediction of second breast screening uptake

	B	SE	Wald	*P*-value	OR	95% CI	Model Accuracy YES	Model Accuracy NO
Model 8: 2nd Screening Uptake							83.8%	65.4%
1st Screening Uptake	-2.278	0.519	19.266	0.000	0.102	0.037, 0.283		
Constant	0.348	0.377	0.853	0.356	1.417			
Model 9: 2nd Screening Uptake							91.9%	61.5%
1st Screening Uptake	2.462	0.571	18.591	0.000	11.728	3.830, 35.914		
Perceived barriers	0.129	0.057	5.102	0.024	1.138	1.017, 1.273		
Breast cancer identity	-0.272	0.123	4.917	0.027	0.762	0.599, 0.969		
Constant	0.157	3.730	0.002	0.966	1.170			

*B* Unstandardized coefficients, *SE* Standard error, *OR* Odds ratio, *CI* Confidence interval

Another model (Model 9) incorporated the Health Beliefs and Illness Perception constructs as covariates, together with the first screening uptake variable as the main independent variable. Model 9 shows that, following the inclusion of all the constructs, ‘Perceived barriers’ and ‘Breast cancer identity’ were found to be important covariates to improve the accuracy of predicting the attendance to the second screening cycle i.e. 83.8% (Model 8) to 91.9% (Model 9). On the other hand, model accuracy dropped from 65.4% (Model 8) to 61.5% (Model 9) when predicting non-attendance to the second invitation at the MBSP.

## Discussion

It is not enough to getting women to initiate BS, but it is essential to encourage them to maintain use over time. Previous studies have examined re-attendance rates [6, 16, 32]. However, BS statistics are not available specifically for a second BS invitation. The available routine BS statistics largely provide cross-sectional estimates of coverage rather than information on women’s ongoing attendance at BS [33]. Moreover, data has long relied on self-reports [34], are measured by area deprivation (using either residential postcodes or general practice postcodes) rather than individual characteristics [35] and can be affected by inflation based on registered general practice lists [33].

Our preliminary rates for Maltese women invited to their subsequent call seem to be lower than those in other countries (Fig. 1: from the total data (*n* = 100), 62% of Maltese women attended the first and second round, 9% had attended the first but not the second round, 17% attended neither call). In a recent study in London among different ethnic groups [6], white British women were most likely to attend their first call (67%) and routine recall (78%). Mixed White and Asian women had the next highest uptake of routine recall invitations (75%), followed by Indian women (first call (61%) or routine recall (74%) appointments), Pakistani (52 and 67%, respectively) and Bangladeshi women (43 and 61%, respectively). The lower subsequent uptake rates in Malta could be due to the programme being relatively new. However, we could not find previous studies that explored re-attendance. We therefore sought to explore the associations and predictive psychosocial factors to second screening based on social cognitive theory.

All sociodemographic characteristics were not significantly associated with second screening uptake and the latter were also found to be non-significant predictors of second screening to the MBSP. These findings are substantiated by similar predictors of returning to a second screen in Australia [16].

More specifically, earlier studies found that women with a breast problem were more likely to undergo clinical breast examination (CBE) and mammography than those who had none [36, 37], while poor self-rated general health status was not associated with ever having had a mammogram in another study [38]. This relationship is likely driven by higher knowledge levels on the benefits of BS [36]. In contrast, we did not observe significant differences between attendees and non-attendees for self-reported health status in this study, except for those who claimed they had a breast condition. The latter women were more likely not to attend for second screening when compared with those who said they did not have any breast problem. We are aware that self-reports may not be as accurate as clinically documented mammographic reports. Nonetheless, in view of the nature and organization of the cognitive and emotional representations of such a health threat, as proposed by CSM [8, 26, 27], women may have opted to attend for private mammography [4], possibly as our earlier study suggests, to obtain an earlier result [7]. Such reasons for non-attendance to second round screening merit further investigation in the local context.

This study also provides evidence that women who were less knowledgeable of the recommended screening frequency were less likely to reattend at the MBSP for a second invitation. There is a similar widespread lack of knowledge of the recommended screening guidelines for a first BS invitation in Malta [4] and in other countries [39, 40]. The impact of the physician-patient relationship may be a reason for the latter finding. In this respect, it is important to note the variation in the order of invitation from service to service. For example, in the UK, women are invited to BS through GP practices [41] whereas in Malta, women are not registered with GP practices but are invited to BS according to age cohorts. Possibly, this infrequent encounter between GPs and Maltese women (Table 1) may be the reason for physicians’ lack of opportunity to address knowledge gaps and to recommend regular BS practices.

Factors associated with BS behaviours in other populations [42, 43] were similar to the second BS round at the MBSP. Namely, Maltese women who believed that they were susceptible to BC, considered the personal consequences of the disease, believed that mammography was not painful, and considered cues to action to motivate them to attend, were more likely than others to re-attend. Similarly, items for BC consequences and cues to action as well as mammography pain were also significantly associated with first BS uptake at the MBSP. Evidence has shown that women’s responsibilities within the family may conflict with self-care and limit screening attendance and re-attendance [44]. Efforts to educate health care providers, particularly physicians, should emphasize the importance of mammography referral and regular physical check-ups [43].

Of the remaining cognitive variables, perceived BC susceptibility and attitude towards use predicted attendance to the second BS round, echoing predictors of repeat use in other studies [45] while contrasting other evidence [32]. Attitudes towards BS behaviour and risk perceptions may be necessary components of why Maltese women contemplate maintaining a behaviour, but may not be prime motivators to influence the initiation of that behaviour.

Self-efficacy was not significantly associated with maintaining BS practices in our study but played a key role in explaining why some women were unable to initiate BS at the MBSP [4]. Although previous research has shown that self-efficacy may be particularly central in moving women from contemplating about undergoing mammography to actually obtaining it [46], in the context of a second BS invitation, the rising challenges or barriers women experience when trying to maintain that behaviour may buffer the intentions that prompt planning for that behaviour [32].

Although several variables differentiated those women who returned for second round screening in the programme from those who did not, the ‘perceived barrier’ construct was found to consistently explain the differences between attendees and non-attendees and to improve the accuracy of predicting attendance to the second screening cycle; a concurrent finding for first invitation to the MBSP [8]. A trial by Farhadifar and colleagues [47] also suggests that regular BS practices are related to fewer barriers. The fact that some women did not return to their second invitation implies that based on their first contact with the screening service, they were less likely to return for routine calls. In the extant literature, this is attributable to a previous negative experience [48], possibly due to pain or discomfort [7, 49], embarrassment, distress, unhelpful staff and/or lower reassurance during the first screen [50]. Our national data also provides evidence that fear of pain was a major factor for not attending the first BS invitation [4] and similarly, this study found pain to be a significant determinant to non-adherence. Preparing women through the invitation letter or in screening campaigns [51] and improving the mammography experience so that it matches women’s expectations could help to increase and maintain BS uptake [6].

The inclusion of both Health Beliefs and Illness Perception items in our study improved the accuracy of predicting non-attendance to the second cycle (Model 6, 84.6%). This finding coincides with findings for first invitation [4]. Given the importance of both theories and the higher predictive accuracy for both BS cycles, it is likely that psychosocial variables, women’s perception of BC and its related risk, and the enactment of cancer control measures predict regular BS behaviours [52] in an attempt to gain control over the disease [53]. This sense of internal control has been proven among disease-effected individuals across diverse health contexts [22, 54, 55] and healthy individuals [8, 52].

Finally, when considering that only one variable was utilised to predict second screening uptake, Model 8 can be considered as an extreme improvement over all the previous logistic regression models (Table 5). Furthermore, the fact that Model 8 utilised only one predictor to predict second uptake makes the model more efficient and easy to use. Moreover, first screening uptake is a very important and significant predictor to predict future uptake as this variable does not require any health beliefs and/or illness perception variables or any other information to predict second BS uptake. Our results further support the evidence that women who obtain at least one mammogram are more likely to obtain subsequent screening [8, 12, 56]. Attendees may be more aware of the possibility for BC to occur, possibly because a BC threat (BC identity, causes and consequences found as CSM predictors for a second BS invitation in this study), coupled by BS benefits, may have helped the individual to ascertain what factors pose barriers to BS attendance and adjust these in the first place. Therefore, what matters most is the social and psychological characteristics, health behaviours and attitudes which women bring to screening the first time, such as a positive attitude towards BS, its value, health behaviours and the belief that an individual would be able to overcome any obstacles to attendance [8, 56]. The most important implication is that if women can be recruited successfully on the first occasion, they will probably stay in the programme. Hence, efforts could focus on identifying and encouraging attendance among women who have never participated in BS and who are reluctant or unsure to participate initially [14] because if women are persuaded to change their beliefs, attendance rates will increase and reattendance will become a matter of routine [17].

### Strengths and limitations

Our study was not subject to response bias because figures for re-attendance were extracted from screening records. This study is one of a few to assess sociodemographic and attitudinal variables as predictors of adherence, but we found none that used initial predictors including HBM and CSM to explore the second BS round. There are some limitations in our research. The limited sample size to examine women’s attendance to a second BS invitation may have reduced the representativeness of the sample. While the percentage of women by district or age was similar to that of the larger study [4], our findings may not be generalizable to the entire Maltese female population invited to their second screening. The characteristics of some women may have changed from first to second screening; this may have introduced misclassification and an underestimation of the relationships presented. Notwithstanding, the study design was necessary for the feasibility to conduct a prospective study to clarify and strengthen our findings, based on an understanding of the culture and attitudes among the Maltese population. Additional research would help to identify barriers and reasons influencing decisions about BS maintenance at the MBSP.

## Conclusions

Researchers have focused more often on promoting behaviour change than on sustaining change. We explored, for the first time, the psychosocial associations, differences and predictors to the second screening invitation based on demographic factors, health status, knowledge, health beliefs, illness perceptions, and actual previous health behaviours. The combination of HBM and CSM variables provided improved prediction of attendance and non-attendance to the second screening call. Perceived barriers, breast cancer identity, causes of BC, and consequences contributed most to the regression models, though perceived barriers was consistently significant across the analyses. Interventions should particularly target non-attendance to first screening. If non-attendees can be persuaded to attend once, they are likely to re-attend, unless their screening experience has been a negative one. The implications of these results are considered for theory, policy and practice to improve the limited understanding of second round screening and to aid the design of culturally sensitive interventions to improve breast screening uptake in Malta.

## Abbreviations

B: Unstandardized coefficients
BC: Breast cancer
BS: Breast screening
CHBMS-MS: Champion’s Health Belief Model Scale for Mammography Screening
CI: Confidence interval
CSM: Common Sense Model
GP: General practitioner
HBM: Health Belief Model
IPQ-R: Revised Illness Perception Questionnaire
M: Mean score
MBSP: Maltese Breast Screening Programme
OR: Odds ratio
SD: Standard deviation
SE: Standard error
